# Cross-national comparisons of increasing suicidal mortality rates for Koreans in the Republic of Korea and Korean Americans in the USA, 2003–2012

**DOI:** 10.1017/S2045796016000792

**Published:** 2016-11-10

**Authors:** A. Kung, K. G. Hastings, K. I. Kapphahn, E. J. Wang, M. R. Cullen, S. L. Ivey, L. P. Palaniappan, S. Chung

**Affiliations:** 1 UC Berkeley - UCSF Joint Medical Program, University of California, Berkeley, California, USA; 2 Division of General Medical Disciplines, Stanford University School of Medicine, Stanford, California, USA; 3 Quantitative Sciences Unit, Stanford University School of Medicine, Stanford, California, USA; 4 Stanford Prevention Research Center, Stanford University School of Medicine, Stanford, California, USA; 5 Population Health Sciences, Stanford University School of Medicine, Stanford, California, USA; 6 School of Public Health, University of California, Berkeley, California, USA; 7 Research Institute, Palo Alto Medical Foundation, Palo Alto, California, USA

**Keywords:** Community mental health, elderly, epidemiology, suicide

## Abstract

**Aims.:**

Korea has the highest suicide rate of developed countries, two times higher than the USA. Suicide trends among Koreans Americans living in the USA during the same period have not yet been described. We report suicide mortality rates and trends for four groups: (1) Korean Americans, (2) non-Hispanic White (NHW) Americans, (3) selected Asian American subgroups and (4) Koreans living in the Republic of Korea.

**Methods.:**

We used US national (*n* = 18 113 585) and World Health Organization (WHO) (*n* = 232 919 253) mortality records for Korea from 2003 to 2012 to calculate suicide rates, all expressed per 100 000 persons. We assessed temporal trends and differences in age, gender and race/ethnicity using binomial regression.

**Results.:**

Suicide rates are highest in Koreans living in the Republic of Korea (32.4 for men and 14.8 for women). Suicide rates in Korean Americans (13.9 for men and 6.5 for women) have nearly doubled from 2003 to 2012 and exceed rates for all other Asian American subgroups (5.4–10.7 for men and 1.6–4.2 for women). Suicide rates among NHWs (21.0 for men and 5.6 for women) remain high. Among elders, suicide in Korean Americans (32.9 for men and 15.4 for women) is the highest of all examined racial/ethnic groups in the USA.

**Conclusions.:**

Suicide in Korean Americans is higher than for other Asian Americans and follows temporal patterns more similar to Korea than the USA. Interventions to prevent suicide in Korean American populations, particularly among the elderly, are needed.

## Introduction

Despite improvements in all-cause mortality, suicide persists as a leading cause of death in the USA, with a rate of 12.4 per 100 000 persons in the past two decades (Heron, 2013). Moreover, the USA exhibits wide racial/ethnic disparities in suicide mortality (Baron *et al.* 2013). The Centers for Disease Control and Prevention (CDC) & National Center for Health Statistics (2013) Health Disparities and Inequalities Report showed that suicide mortality is highest among American Indian/Alaska Natives and non-Hispanic Whites (NHWs) and relatively low in non-Hispanic Blacks, Asian/Pacific Islanders and Hispanics (Baron *et al.* 2013). However, these racial/ethnic groups obscure mortality trends within highly heterogeneous populations, particularly for Asian Americans (Holland & Palaniappan, 2012).

Contrary to the expected low suicide mortality among Asian Americans at the national level, there is wide variation among Asian American subgroups. Among the six largest subgroups – Asian Indian, Chinese, Filipino, Japanese, Korean and Vietnamese – the frequency of suicide among Korean Americans is exceptionally high (Hastings *et al.* 2015). By comparison, the rate of suicide mortality in the Republic of Korea (hereafter: Korea) has nearly tripled from 1990 to 2010, and is the highest of all developed countries (OECD, 2013). Given that 73% of Korean Americans are foreign-born (U.S. Census Bureau, 2013), it is possible that similarly high rates of suicide may be observed among Korean Americans. In Korea, studies have cited increasing income inequality, the weakening of social solidarity, and social isolation and poverty among the elderly as potential contributing factors to high suicide rates (Kwon *et al.* 2009). Altogether, these trends have coincided with high rates of suicide mortality for both countries – Korea had 28.5 suicides per 100 000 population in 2013 (Korean National Statistical Office, 2013), while the USA experienced roughly half that, or 13.0 per 100 000, in that same year (Centers for Disease Control and Prevention (CDC) & National Center for Health Statistics, 2013).

Current knowledge on suicide within Asian American populations is inadequate and imprecise, and researchers have specifically called for the disaggregation of Asian American suicide statistics by ethnic subgroup as an urgent priority and challenge (Han *et al.* 2013). Besides the methodological challenges of collecting health data on Asian American subgroups, suicide mortality is particularly difficult to assess due to sociocultural reasons, underreporting and potential misclassification (O'Carroll, 1989). In the absence of data on suicide mortality, a few studies have sought to understand suicidal behaviours among Asian American subgroups (Duldulao *et al.* 2009; Cheng *et al.* 2010; Wong *et al.* 2013). However, within this research, the sample sizes for Korean Americans are small (*n* = 127 for the largest study), preventing both a comprehensive descriptive analysis of Korean Americans and a detailed comparative understanding of suicide in Korean Americans relative to other Asian American subgroups. Research on suicidal behaviours in Asian American subgroups have largely been based on the National Latino and Asian American Study (NLAAS), where undersampling of Korean Americans necessitated their data be subsumed under the category of ‘other Asian’ (Duldulao *et al.* 2009; Cheng *et al.* 2010). Research on suicide prevention has also neglected to include Korean Americans.

Characterising suicide mortality in Korean Americans is of particular importance because studies have shown Korean Americans bear a disproportionate burden of suicide risk factors. In one study, California Health Interview Survey (CHIS) data found that Koreans were 2.1 times more likely than NHWs to report symptoms indicative of mental distress (Sorkin *et al.* 2011). A meta-analysis and systematic review also revealed that rates of depression, one of the most important risk factors for suicide, were twice as high among Korean Americans as Chinese Americans (Kim *et al.* 2015). Korean Americans were also more likely to report suicidal ideation, at three times the rate of other Asian American subgroups (Wong *et al.* 2013).

Recent work on disaggregating leading causes of death for Asian Americans show that for Korean Americans, these increased suicide risk factors may indeed result in fatal consequences. One in every 20 deaths for Korean American men is due to suicide, compared with roughly one in 40 for NHW men (Hastings *et al.* 2015). By comparison, the frequency of suicide for other Asian American subgroups was 2.9% for Asian Indian men, 1.8% for Chinese men, 1.6% for Filipino men and 2.9% for Vietnamese men (Hastings *et al.* 2015). It is clear from these data that Korean Americans carry a far greater relative burden of suicide. However, the absolute burden of suicide, as understood through suicide mortality rates, has yet to be established for Korean Americans and other Asian American subgroups at the national level.

Moreover, suicide mortality trends within Asian American subgroups by age and gender have yet to be adequately characterised. These data are important, as knowing which populations are most at risk are essential for developing and implementing targeted, effective interventions to prevent suicide. Suicide mortality rates exhibit patterns by gender in suicide methods (Park *et al.* 2014*a*; Hee Ahn *et al.* 2012), and prior work has also shown suicide to particularly affect the elderly in Korea (Kwon *et al*. 2009). Understanding whether such trends are also evident in the Korean American population may provide some insight on how immigration affects suicide mortality rates for Koreans while also informing tailored interventions for Korean Americans.

Our study fills existing gaps in the literature by performing a cross-national comparison of suicide mortality rates between Korean Americans, NHW Americans, selected Asian American subgroups and Koreans in Korea. Our mortality data allow us to report suicide as a cause of death, but does not provide information on suicidal ideation or suicide attempts. We hypothesise that given the recent immigration history of Korean Americans, Korean Americans will show suicide mortality patterns more similar to Koreans than to NHWs in the USA. To test this hypothesis, we report national suicide mortality rates for Koreans in Korea, NHWs in the USA and disaggregated Asian American subgroups in the USA by age, gender and race/ethnicity.

## Methods

### Study population

We used data from the World Health Organization (WHO) to determine population and absolute death counts in Korea (WHO Mortality Database, 2015). The WHO data for Korea comes from the Korean National Statistical Office, which has 100% civil registration coverage of cause of death for Korea. We included all deaths whose underlying cause was attributed to suicide based on 10th revision of the International Statistical Classification of Diseases and Related Health Problems (ICD-10) codes (X60-X84, X87) in the years 2003–2012.

Mortality data for US racial/ethnic groups came from the National Center for Health Statistics’ (NCHS) Multiple Cause of Death mortality files. We used the race, gender and age-specific linear interpolation between the 2000 and 2010 Censuses to define our year-specific reference populations. We started the study period at 2003 as this was the first year that states began to disaggregate the six major Asian subgroups (Asian Indian, Chinese, Filipino, Japanese, Korean, Vietnamese) using the 2003 revision of the standard US death certificate.

The data include 36 states that adopted the 2003 revision of the US Standard Certificate of Death, which allows for the disaggregation of Asian American decedents into the following racial/ethnic subgroups: Asian Indian, Chinese, Filipino, Japanese, Korean, or Vietnamese. Eighty per cent of the total Korean American population is represented in these 36 States (not included: AL, AK, CO, HI, LA, MD, MA, MS, NC, PA, TN, VA, WV, WI), according to the 2010 Census. Decedents that reported as more than one Asian race, Asian Hispanic, or ‘other Asian’ were excluded from the current analysis. As with the Korean data, we included only deaths whose underlying cause was attributed to suicide based on ICD-10 codes (X60-X84, X87) in the years 2003–2012.

All the data sources for the study (NCHS Multiple Cause of Death mortality files, 2000 and 2010 US Census data, WHO Mortality Database) are publicly available and analytical data can be shared as per request.

### Statistical analysis

**Fig. 1.**
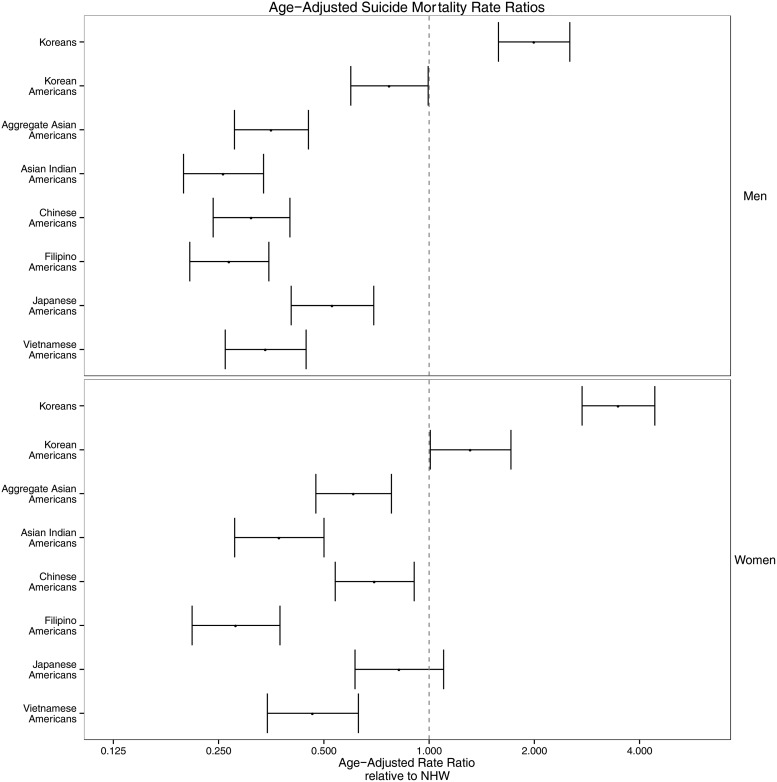
Age-adjusted suicide mortality rate ratios in Korea and selected Asian American subgroups with Non-Hispanic Whites (NHWs) as the referent group. The *x*-axis is on the log scale.

**Fig. 2.**
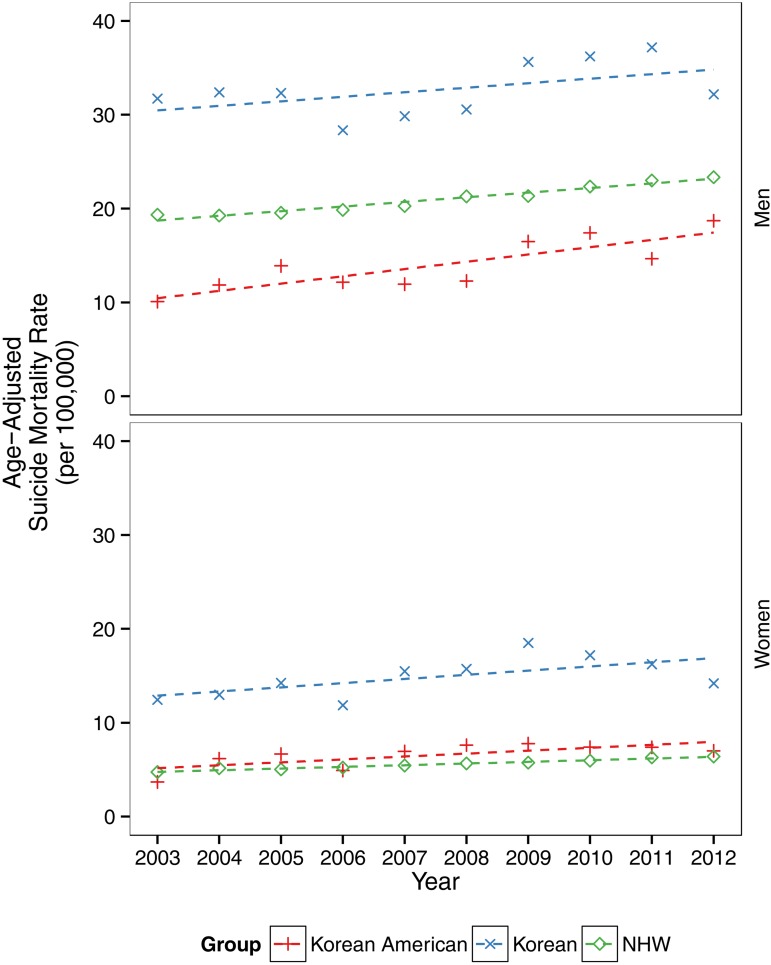
Temporal trends in suicide mortality rates in Korea, Korean Americans and non-Hispanic Whites (NHWs) in the USA, 2003–2012.

Methods of how US mortality data were calculated are discussed in our previous analysis (Hastings *et al.* 2015). In summary, age-adjusted suicidal mortality rates per 100 000 population were calculated using direct standardisation using the 2000 WHO standard population for both the USA and Korea and are presented in Table 1. We adjusted for age in two ways for these analyses. We used direct standardisation to adjust for age in our point estimates of race and gender specific mortality rates (i.e., in Table 1 and Fig. 2). We also used regression models as a basis of inference for determining statistically significant differences in mortality rates between different subgroups of interest. For the regression models, age-adjustment refers to the inclusion of age as a covariate in the regression model (i.e., in Table 2 and Fig. 1). We used negative binomial logistic regression to estimate race/ethnicity specific age-adjusted mortality rates and rate ratios. These models also included the natural logarithm of population as an offset.

**Table 1.** Total number of deaths, age-adjusted all-cause mortality rates and age-adjusted suicide mortality rates for Korean (WHO data), Korean American, Non-Hispanic White (NHW), aggregate Asian American and selected Asian American subgroups, 2003–2012. (36 state data)

	Koreans	Korean Americans	Non-Hispanic Whites (NHWs)	Aggregate Asian Americans^a^	Asian Indian Americans	Chinese Americans	Filipino Americans	Japanese Americans	Vietnamese Americans
Men									
Population size	24 977 164	514 594	70 846 958	4 820 509	1 204 010	1 328 769	939 162	214 807	619 167
Total number of deaths, 2003–2012	1 377 742	14 490	7 076 443	150 284	22 164	47 065	34 691	16 253	15 621
Age-adjusted all-cause mortality rate	609.9	299.3	592.1	298.8	274.0	268.7	347.5	336.3	281.4
Age-adjusted suicide mortality rate	32.4	13.9	21.0	6.9	5.4	5.4	5.9	10.7	7.6
Women									
Population size	24 902 648	624 865	73 110 261	5 386 009	1 115 216	1 505 514	1 187 590	300 157	652 667
Total number of deaths, 2003–2012	1 109 632	16 302	7 351 928	142 506	15 198	42 337	34 988	21 663	12 018
Age-adjusted all-cause mortality rate	325.0	218.9	406.0	203.4	198.4	184.7	213.4	230.9	192.5
Age-adjusted suicide mortality rate	14.8	6.5	5.6	2.9	1.9	3.0	1.6	4.2	2.4

a Aggregate Asian Americans combine data from Asian Indian, Chinese, Filipino, Japanese, Korean and Vietnamese Americans.

**Table 2.** Age-adjusted mortality rates for Korea, Korean American, Non-Hispanic White (NHW), aggregate Asian American and selected Asian American subgroups by sex and age group, 2003–2012.

	Koreans	Korean Americans	Non-Hispanic Whites (NHWs)	Aggregate Asian Americans^a^	Asian Indian Americans	Chinese Americans	Filipino Americans	Japanese Americans	Vietnamese Americans
Men									
Age 0–19	4.2 (3.3, 5.0)	2.2 (0.0, 4.4)	5.2 (4.2, 6.1)	1.6 (0.9, 2.3)	1.2 (0.1, 2.4)	0.9 (0.0, 1.9)	2.6 (0.7, 4.5)	3.6 (0.0, 8.9)	1.5 (0.0, 3.1)
Age 20–34	34.0 (27.8, 40.1)	17.2 (10.3, 24.1)	28.3 (23.2, 33.3)	10.3 (7.8, 12.8)	8.6 (5.2, 11.9)	6.7 (3.9, 9.4)	11.2 (6.4, 15.9)	14.1 (4.2, 24.1)	15.1 (8.2, 22.0)
Age 35–49	39.9 (32.8, 47.1)	20.3 (12.3, 28.2)	42.0 (34.5, 49.5)	10.0 (7.5, 12.4)	7.7 (4.3, 11.1)	7.2 (4.4, 10.0)	8.6 (4.6, 12.6)	16.9 (7.2, 26.6)	11.0 (5.8, 16.3)
Age 50–64	53.1 (43.5, 62.7)	28.4 (17.1, 39.8)	39.5 (32.5, 46.5)	11.6 (8.6, 14.6)	8.6 (4.0, 13.1)	9.7 (5.9, 13.5)	7.1 (3.2, 10.9)	19.9 (8.3, 31.4)	10.4 (4.2, 16.6)
Age 65+	114.5 (94.0, 135.1)	32.9 (17.6, 48.3)	29.0 (23.8, 34.2)	14.8 (10.7, 19.0)	7.7 (1.6, 13.8)	18.3 (11.3, 25.2)	5.3 (0.9, 9.7)	17.9 (5.9, 29.9)	8.6 (1.0, 16.3)
Women									
Age 0–19	2.1 (1.7, 2.5)	1.0 (0.0, 2.1)	1.3 (1.0, 1.5)	0.7 (0.4, 1.0)	0.4 (0.0, 0.9)	0.5 (0.0, 1.0)	0.7 (0.1, 1.2)	1.4 (0.0, 3.6)	0.5 (0.0, 1.0)
Age 20–34	17.1 (14.0, 20.2)	8.0 (4.5, 11.6)	6.9 (5.7, 8.1)	4.2 (3.1, 5.4)	3.0 (1.5, 4.5)	3.7 (2.0, 5.3)	2.8 (1.2, 4.4)	5.5 (1.2, 9.8)	4.7 (1.9, 7.5)
Age 35–49	20.1 (16.5, 23.7)	9.4 (5.4, 13.4)	10.2 (8.4, 12.1)	4.1 (3.0, 5.2)	2.7 (1.2, 4.2)	4.0 (2.3, 5.6)	2.2 (0.9, 3.4)	6.6 (2.3, 11.0)	3.4 (1.3, 5.5)
Age 50–64	26.7 (21.9, 31.5)	13.3 (7.6, 18.9)	9.6 (7.9, 11.3)	4.8 (3.5, 6.1)	3.0 (1.1, 5.0)	5.3 (3.1, 7.6)	1.8 (0.6, 2.9)	7.8 (2.6, 13.0)	3.2 (0.9, 5.5)
Age 65+	57.6 (47.2, 67.9)	15.4 (7.8, 22.9)	7.1 (5.8, 8.3)	6.1 (4.3, 8.0)	2.7 (0.4, 5.1)	10.0 (5.9, 14.1)	1.3 (0.1, 2.5)	7.0 (2.1, 11.9)	2.7 (0.1, 5.3)

a Aggregate Asian Americans combine data from Asian Indian, Chinese, Filipino, Japanese, Korean and Vietnamese Americans.

To estimate age-adjusted suicide rate ratios, we fit negative binomial regression models with number of suicide deaths as the dependent variable and race/ethnicity, age, gender and race/ethnicity-gender as the interaction term (Fig. 2).

We also fit negative models with number of suicide deaths as the dependent variable and race/ethnicity, age, gender, an age–race interaction term, a race/ethnicity–gender interaction and an age–gender interaction term. These models allow for assessment of differences between age, race/ethnicity and gender via Wald tests on the interaction terms, and also allow estimation of age group specific age-adjusted mortality rates (Table 2 and Fig. 1).

Trends were analysed by fitting gender-specific negative binomial models with number of suicide deaths as the dependent variable and year, race, age and a year–race interaction term (Fig. 2).

## Results

The study populations consisted of 2 487 374 decedents in Korea and 18 113 585 decedents from 36 states in the USA from 2003 to 2012. Korea presented the highest suicide mortality rates of all the groups studied. The average suicide mortality rate between 2003 and 2012 for Korea was 32.4 for men and 14.8 for women. In comparison, NHWs had the highest rate of suicide among men (21.0) in the USA while Korean Americans had the highest rate of suicide among women (6.5) in the USA. Korean Americans had the highest suicide mortality rates of all Asian American subgroups (13.9 for men, 6.5 for women), which were roughly twice the rates of aggregate Asian Americans (6.9 for men, 2.9 for women).

Male-to-female suicide ratios were more similar between Koreans (2.2) and Korean Americans (2.1) than NHWs (3.8). Assessment of differences via regression also showed a statistically significant difference in age-adjusted suicide mortality, with higher rates seen in Korea compared with rates in Korean Americans for both men (*p* < 0.001) and women (*p* < 0.001).

Suicide mortality rates differed significantly across racial/ethnic groups (*p* < 0.001), gender (*p* < 0.001) and age groups (*p* < 0.001) (Table 2). In Korea, the highest rates of suicide were among age >65 year old men (114.5), which were roughly double that of the next highest groups: >65 year old women (57.6) and 50–64 year old men (53.1). In the USA, the groups with the highest suicide rates among men were 35–49 year old NHWs (42.0), 50–64 year old NHWs (39.5) and >65 year old Korean Americans (32.9). For women in the USA, >65 year old Korean Americans (15.4) had the highest rates of suicide, followed by 50–64 year old Korean Americans (13.3) and 35–49 year old NHWs (10.2). Elderly (>65 year old) Korean American men and women have the highest suicide mortality rates of all Asian American subgroups by gender and age.

To compare suicide rates in Koreans and selected US racial/ethnic groups, we used suicide rates among NHWs in the USA as the referent group to show suicide mortality rate ratios (Fig. 1). The groups that exceed NHW suicide rates are Korean men, Korean women and Korean American women.

There was a general linear increase in age-adjusted suicide mortality rates across all comparison groups: Koreans, Korean Americans and NHWs (Fig. 2). Deviation from the linear trend (fall in 2006 and rise in 2009) is similar between Koreans and Korean Americans. This temporal pattern does not appear among NHWs in the USA.

Suicide mortality rates for Korean American men (increasing from 10.1 to 18.7; 85% change) and women (increasing from 3.7 to 7.0; 90% change) nearly doubled from 2003 to 2012. In comparison, suicide mortality rates increased by 1% for Korean men (31.7–32.2), 14% for Korean women (12.4–14.2), 20% for NHW men (19.3–23.3) and 35% for NHW women (4.7–6.4) over the study period. The differences in rate of increase among the three comparison groups (Koreans, Korean Americans and NHWs) are not statistically significant.

## Discussion

To our knowledge, no other study has reported national suicide mortality rates for Korean Americans and other Asian American subgroups and prior to 2003 such descriptive analysis was not possible. Our study shows that Koreans in Korea and NHWs and Korean Americans in the USA are at particular risk for suicide deaths compared with other Asian American subgroups in the USA. For Korea, the average adjusted suicide mortality rates from 2003 to 2012 were 32.4 per 100 000 for men and 14.8 for women, which significantly exceed the Organization for Economic Co-operation and Development (OECD) average of 20.78 for men and 5.92 for women in 2010 (OECD, 2013). There is no evidence to our knowledge that suggests over reporting of suicide as a cause of death in Korea compared with other OECD countries. It is more likely that suicide is under-reported due to stigma (Khang *et al*. 2005). Furthermore, most (>90%) of deaths are verified by physicians in Korea (Jo *et al*. 2004), which likely increases the fidelity of the data. Meanwhile, Korean Americans had the highest suicide mortality rates of all Asian American subgroups, and the Korean American elderly (>65 years old) and Korean American women had suicide mortality rates even higher than did NHWs. Of the three comparison groups, Korean Americans experienced the greatest increase in suicide mortality from 2003 to 2012 (85% for men and 90% for women).

The potential reasons behind these high suicide mortality statistics are complex and multifactorial. In Korea, authors, journalists and researchers have postulated that rapid economic development and uncertainty, high expectations for achievement and continued stigma around mental illness may drive Korea's alarmingly high rates of suicide (Kim, 2014; Al Jazeera English, 2015). More specifically, Korea's gross domestic product increased 60-fold from the 1960s to the present day (Kwon *et al.* 2009). Such rapid economic growth may have exposed a tension between older collectivistic Confucian ideals and newer individualistic and capitalistic values, manifesting as social and psychological distress that could contribute to suicide, particularly among elderly (Park, 2013). Research has also shown that in Korea, factors such as low public social expenditure (Park *et al.* 2009), unemployment (Inoue *et al.* 2010), low job control (Yoon & Chang, 2014), sleep problems (Kim *et al.* 2013), lower academic performance (Kang *et al.* 2014) and weight-related attitudes and behaviours (Kim & Lee, 2010) demonstrate positive relationships with suicide risk. Korea also appears to be unique in its frequency of high-profile suicides that result in waves of copycat suicides (Suh *et al.* 2015). In copycat suicides, specific methods of suicide, such as pesticide poisoning, charcoal-burning (Lee *et al.* 2014*a*), hanging, jumping, or other methods (Park *et al.* 2014*b*) can be reported in the news and are often replicated shortly after being reported.

According to one major hypothesis, ‘For immigrant groups, in general, suicide rates tend to mirror the rates in the country of origin and converge toward the rate in the host country over time,’ (Jacobs *et al.* 2003). Korean Americans are the second largest expatriate Korean community in the world and the majority (73%) of the Korean American population is foreign-born (U.S. Census Bureau, 2013). We would therefore expect the high suicide rates among Koreans to persist among Korean Americans and potentially decrease over time. Our findings present a mixed picture. Rates of suicide mortality for Korean Americans are less than half the rates of their Korean counterparts, indicating the possibility that those who immigrate have lower risk factors for, and/or greater protective factors against, suicide relative to Koreans. Suicide rates for Korean Americans have indeed more closely mirrored trends of increasing suicide in Korea instead of decreasing. However, Korean American suicide rates also differ from Korean rates in the degree of increase in suicide rates, which have nearly doubled over the study period (2003–2012) for Korean Americans. Finally, it is difficult to assess whether rates of suicide for Korean Americans appear to be converging to the US average, which is largely driven by NHWs. While the Korean American suicide mortality rate is closer to that of NHWs than Asian American subgroups, it is unlikely that their experience in the USA is closer to that of NHWs than other Asian American subgroups.

Our case study of reporting high suicide mortality rates is also unexpected, given prior literature that suggests that immigrants have lower risk for mood disorders (Salas-Wright *et al.* 2014). Moreover, such risk may be informed by nativity and amount of time spent in the USA – data from the aforementioned NLAAS study shows that for Asian Americans, US-born women bore the greatest risk for any mood disorders (Hong *et al.* 2014), and for U.S. Latinos, ‘immigrants who arrived in the USA after early childhood experience[d] significantly lower risks of depression, anxiety and substance-use disorders in their country of origin compared with US-born Latinos of the same age and sex,’ (Alegria *et al.* 2007). Although some work has framed these seemingly low rates of mental health burden among immigrants as an ‘immigrant health paradox’, most studies agree these population statistics are the reflection of multiple complex and possibly interacting, phenomena (John *et al.* 2012; Lau *et al.* 2013; Ro, 2014). We were unable to explore this further as our data did not include a variable for nativity or number of years in the USA. However, our findings of high rates of suicide mortality among Korean Americans, which may have otherwise been obscured by broad categories such as ‘Asian’ or ‘immigrant’, highlight the shortcomings of using aggregated categories to characterise mental health and mortality burden.

Our results also bring attention to a notable gap in descriptive analysis of Korean Americans in prior literature. Little research specific to Korean Americans exists that describes potential drivers for suicide to help explain how they have the greatest absolute burden of suicide of all Asian American subgroups. Meanwhile, research in Asian Americans more broadly have shown that Korean Americans experience twice the rate of depression of Chinese Americans (Kim *et al.* 2015) and the highest rate of suicidal ideation among Asian American subgroups (Wong *et al.* 2013). Korean Americans also have the highest rates of alcohol use among Asian American subgroups (Lee *et al.* 2013), which has been linked with increased suicide risk (Groves *et al.* 2007). Protective factors against suicide appear to be fewer and less well documented, although high ethnic identification or pride among recent Korean American immigrants may buffer against depression and suicidal ideation (Choi *et al.* 2009). These findings suggest that suicide risk factors outweigh protective factors for this population and may contribute to the high suicide mortality rates seen in Korean Americans as evidenced in our study.

Moreover, studies also show that Korean Americans are not accessing needed care. Even after adjusting for health characteristics and English-language proficiency, Korean Americans were less likely to have seen a primary care provider compared with NHWs (Sorkin *et al.* 2011). Mental health utilisation is also low among Korean Americans (Jang *et al.* 2007) and may be partially explained by the substantive barriers Korean Americans face, which include having the lowest rate of health insurance among Asian American subgroups (Yu *et al.* 2010), and a high degree of stigma surrounding mental illness (Bernstein, 2007). Korean Americans may delay or avoid care as a result, which is reinforced by the belief that depression can be overcome with endurance, patience and religion (Bernstein, 2007). The failure to see the need for psychological help has also been documented in Korean American communities, which has been noted as a major reason why Korean Americans do not utilise mental health services (Park *et al.* 2013). More peripheral research also suggests that sociocultural factors may play a role. As previously mentioned, the majority (73%) of the Korean American population is foreign-born (U.S. Census Bureau, 2013), and accompanying language barriers (Gee & Ponce, 2010), acculturative stress (Oh *et al.* 2002) and discrimination (Noh & Kaspar, 2003) have each been shown to have negative effects on health and mental health, which could contribute to increased suicide.

This study also showed heterogeneity of suicide mortality rates across age groups, with high rates of suicide among Korean and Korean American elderly. For Korea, rapid economic change may also play a role in this disproportionate effect on the elderly. In Korea, the near-elderly face a compulsory retirement age at 50–66 years of age, while their children migrate to urban areas (Korean National Bureau of Statistics, 2013). Nearly half of Korean elders (46.1%) live in relative poverty with minimal welfare protection from the government and few sources of social support (Organization for Economic Co-operation and Development, 2015). Meanwhile there is emerging evidence that in the USA, elderly Korean Americans experience severe psychological distress and isolation (Park *et al.* 2015). These factors may help explain the high suicide rates in our findings, with rates of 114.5 for Korean >65 year old men, 57.6 for Korean >65 year old women, and 32.9 and 15.4 for Korean American >65 year old men and women, respectively.

Koreans and Korean Americans may also be vulnerable to economic change in similar ways. Following the 1998 financial crisis in Korea, there was a peak in suicide mortality (Khang *et al.* 2005; Chang *et al.* 2009); our data demonstrate an increase in suicide mortality in 2009 following the 2008 global economic crisis. In Korea, financial problems were frequently the precipitating event to suicide for men (Im *et al.* 2011), and in the USA, financial hardship has a significant correlation with perceived well-being and vitality for Korean American immigrants as well (Lee & Woo, 2013). It is possible that a disproportionate portion of suicide deaths among Koreans and Korean Americans documented in our study occurred among individuals experiencing economic hardship, but we were unable to ascertain this with our study variables.

Overall, our results are consistent with prior work showing differential patterns in suicide rates between Asian and Western countries. Notably, Koreans and Korean Americans have trends consistent with Asian countries at a broad level, where there are generally higher average suicide rates and lower male-to-female gender ratios in suicide deaths (Chen *et al.* 2012). Our study found 2.2 male-to-female ratios in suicide mortality among Koreans from 2003 to 2012, which approximates the 1.8 male-to-female ratio in suicide mortality in Koreans shown in a study from 2000 to 2009 (Hee Ahn *et al.* 2012). This lower male-to-female suicide ratio compared with NHWs and Western countries may be partially explained by gender-specific differences in suicide methods such as hanging, which is more common among women than men in Korea (Hee Ahn *et al.* 2012), but does not necessarily help explain the high suicide rates among Korean American women, whose suicide methods are unknown. In contrast, NHWs had a 3.8 male-to-female gender ratio in our study, which is the same ratio found in suicide deaths for the USA overall in prior work (Chen *et al.* 2012). This similar male-to-female gender ratio in suicide suggests that trends in the NHW population, who has the greatest numbers of suicides, drive most of aggregated national suicide statistics. With our findings, suicide among Korean and Korean American women emerges as an important and possibly underappreciated phenomenon.

Our study demonstrates the potential for comparative work and sociocultural context to help shed light on cause-specific standardised mortality rates in racial/ethnic subgroups. For example, because Korean Americans are a largely foreign-born population, a comparative context that includes population health statistics and sociocultural dynamics in the country of origin, immigration histories and trends, and unique experiences in the USA may be appropriate in helping explain mortality patterns. This contextual approach may also serve to help shed light on the potential under-diagnosis and detection of mental health issues among various Asian American subgroups. Population surveys rarely include Asian Americans with limited English proficiency, and rates of self-report are likely limited by the high level of cultural stigma associated with mental illness among Asians.

The findings from this study have several implications for population health, research and practice. First, on the population-level, our study demonstrates the need for more research on the drivers behind suicide rates, particularly among Koreans, Korean Americans and NHWs. Known risk factors for suicide include depression and suicidal ideation, which need to be more accurately and systematically assessed. Moreover, these methods of assessment could consider accounting for differential manifestations of mental distress by Asian patients, such as through somatisation or culture-bound syndromes such as *hwabyung*, a diagnosis seen most commonly among Koreans. These manifestations may mask the true prevalence of mental disorders (Park *et al.* 2012) and could affect accurate assessment of suicide risk.

Second, this study highlights the need for continued research on suicide prevention. In the USA, these efforts are particularly needed for ages 35–49 and 50–64 for NHWs (Caine, 2013), and ages 50–64 and >65 for Korean Americans, where we see the highest rates of suicide. Evaluations of mental health interventions (Lee *et al.* 2014*b*) that reduce isolation and depression could help build effective, evidence-based programs that can mitigate these increasing suicide rates. Health system interventions such as creative integration of mental health services into primary care and social service delivery, and enrolment into affordable insurance products with parity in mental health coverage may also have implications for reducing suicide risk and are worthy of investigation. In addition, prevention research could consider assessing the effectiveness of restricting access to lethal means of suicide, such as firearms. Moreover, current clinical practice protocols in the USA could also benefit from acknowledging the increased risk of suicide for Korean Americans.

Finally, our study demonstrates that Koreans living in the USA have much lower suicide rates than Koreans living in Korea. Therefore, suicide may be better understood primarily as a problem at the social level rather than a problem at the individual level. The interventions and policies that follow should consequently focus specifically on population-level interventions in addition to individual-level, or clinical, approaches for decreasing suicide rates in Korea.

A few limitations are worth mentioning. The possibility of misclassification of race/ethnicity within the death certificates is plausible (National Center for Health Statistics & Centers for Disease Control and Prevention (CDC), 2001). Prior research has shown that the minority populations may be more prone to misclassification for suicide than NHWs (Rockett *et al.* 2010). Misclassification may over or underrepresent calculated rates but sensitivity analyses in a previous study suggest rates are nominally affected (Hastings *et al.* 2015). In the WHO data, unclear causes of death (ICD-10 Codes R0-R99) accounted for 5.6% of the Korean death data, but only 1.5% for the USA mortality data. It is possible that the data underestimate suicide mortality rates due to reporting discrepancies (Park *et al.* 2014*a*), and more severely so in Korea than in the USA. Of the 50 states, 36 have adopted the 2003 standard death certificate to report disaggregated Asian Americans, but these same states adopted the standard in different years throughout the time period (2003–2012). Prior sensitivity analyses have confirmed that rates from 36 state data are not statistically different from 50 state data (Hastings *et al.* 2015). Mortality records do not include variables such as acculturation, comorbidities, mental illness, alcohol use, family history and other important demographic factors that have been shown to be associated with suicide rates. Another limitation is that this study only reports suicide mortality rates and is unable to provide information on suicidal ideation and suicide attempts.

Despite evidence pointing towards a mental health burden experienced by Korean Americans, no study to our knowledge has comprehensively described suicidal mortality for this population. Prior literature has lacked the scope and statistical power needed to establish Korean American suicide as a public health issue. Our results show rates of suicide for Koreans are high, and rates for Korean Americans are higher than for other Asian subgroups but lower than for NHW men. These findings illustrate the importance of disaggregating Asian American health statistics, which uncovered high rates of suicide as an issue among Korean Americans. Our results also suggest that more research should be done in mental health, suicide risk and suicide prevention to improve targeted public health agendas, especially as Asian American populations increase over time in the USA. Suicidal mortality of women and elderly Koreans and Korean Americans is particularly higher than other groups, suggesting certain cultural factors may contribute to these phenomena. Greater understanding of contributors of their higher risk of suicide and tailored interventions for these populations in particular are necessary.

## Availability of Data and Materials

All data underlying the authors' findings in this study are freely available in public repositories, regulated by the National Center for Health Statistics (NCHS) and the World Health Organization. The authors have obtained individual county-level characteristic data from NCHS for the analyses, thus its public use is restricted. If interested in requesting these data, please visit the following link for more information: http://www.cdc.gov/nchs/data_access/data_linkage/mortality/data_files_data_dictionaries.htm.
